# Patient’s quality of life after surgery and radiotherapy for extremity soft tissue sarcoma - a retrospective single-center study over ten years

**DOI:** 10.1186/s12955-019-1236-4

**Published:** 2019-11-08

**Authors:** Rebekka Götzl, Sebastian Sterzinger, Sabine Semrau, Nikolaos Vassos, Werner Hohenberger, Robert Grützmann, Abbas Agaimy, Andreas Arkudas, Raymund E. Horch, Justus P. Beier

**Affiliations:** 1 Department of Plastic and Hand Surgery, Comprehensive Cancer Center, Universitiy Hospital of Erlangen, Friedrich-Alexander-University Erlangen-Nürnberg (FAU), Erlangen, Germany; 2 Present Address: Department of Plastic Surgery, Hand and Burn Surgery, University Hospital of Aachen, RWTH University of Aachen, Aachen, Germany; 3 Department of Radiation Oncology, Comprehensive Cancer Center, University Hospital of Erlangen, Friedrich-Alexander-University Erlangen-Nürnberg (FAU), Erlangen, Germany; 4 Department of Surgery, Comprehensive Cancer Center, University Hospital of Erlangen, Friedrich-Alexander-University Erlangen-Nürnberg (FAU), Erlangen, Germany; 5 Department of Pathology, Comprehensive Cancer Center, University Hospital of Erlangen, Friedrich-Alexander-University Erlangen-Nürnberg (FAU), Erlangen, Germany

**Keywords:** Soft tissue sarcoma, QLQ-C30, Sarcoma radiotherapy, Sarcoma major complication rates, Health related quality of life

## Abstract

**Background and objectives:**

The purpose of this study is to analyze major complication rates and different aspects of health-related quality of life (HRQoL) in extremity soft tissue sarcoma (STS) patients treated with or without radio (chemo) therapy and surgery.

**Methods:**

We performed a retrospective analysis of all patients who underwent Extremity STS excision from 2004 to 2014 (182 patients included). Patients’ data were collected from patients’ records. HRQoL was assessed by using EORTC QLQ-C30.

**Results:**

A total of 182 patients underwent sarcoma resection. After neoadjuvant radiochemotherapy (RCT), the major-complication rate amounted to 28% (vs. 7%, no radiotherapy, *p* <  0.001). Major-complication rates after adjuvant radiotherapy (RT) occurred in 8% (vs. 7%, no radiotherapy, *p* = 0.265). Comparison QoL scores between treating with neoadjuvant RCT or without RT revealed significant worse scores with neoadjuvant RCT. Further stratification of disease control of these patients showed significant reduced scores in the group of disease-free patients with neoadjuvant RCT compared to irradiated disease-free patients.

**Discussion:**

To date, there have only been a few investigations of QoL in STS. Retrospective study on quality of life have limitations, like a lack of baseline evaluation of QoL. Patient candidated to radiation therapy could have had worse QoL baseline due to more advanced disease. Disease status of the patients who answered the questionnaires could have been an influence of QoL and we could show reduced scores in the group of disease-free patients with neoadjuvant RCT, but not for the patients with recurrence or metastasis, so it is very hard to discriminate whether radiation therapy could really have an impact or not.

**Conclusion:**

This study might assist in further improving the understanding of QoL in STS patients and may animate for prospective studies examining the oncological therapies impact on HRQoL.

## Background

Soft tissue sarcomas are rare and account for only < 1% of all malignancies. Modern sarcoma treatment is an interdisciplinary challenge. Besides surgery, playing the central role, radiotherapy and chemotherapy are other important treatment modalities. Historically, radical surgery including limb amputation was often used to achieve wide longitudinal margins. Studies performed in the 1970s and 1980s showed no influence on overall survival, when limb-conserving surgery and adjuvant radiotherapy are combined, compared with radical amputation alone [1].

Prospective and retrospective studies have suggested that radiotherapy improves the local control rates in the setting of resectable disease and the overall survival [2, 3]. But, there is still a lack of survival impact of RT in STS, especially in extremity STS. Newer retrospective analyses showing an impact by RT on survival in STS are largely open to criticism [4]. However, many authors describe that wound complication rates are higher after preoperative irradiation, and long-term function is worse after postoperative irradiation, probably as a result of higher postoperative radiation doses, larger radiation fields, and resulting fibrosis [5, 6].

Multimodal treatment regimens might inflict a substantial morbidity and mortality with a substantial effect on health-related quality of life (HRQoL). When counseling patients on various treatment options, information of quality of life (QoL) following various types of treatment is therefore very important [7].

For a long time, soft tissue sarcoma patients had poor 5-year survival rates below 50%. With improvements in diagnosis and treatment, 5-year survival rate increased to 60–70% [5]. More patients with soft tissue sarcoma became long-term survivors and analysis of HRQoL should be much more important. However, little attention has been paid to this issue in the literature [8]. In particular, little is known about the role of RT for QoL in soft tissue sarcoma treatment.

The purpose of this study is to analyze different aspects of HRQoL in soft tissue sarcoma patients treated with or without radiotherapy and surgery over a period of 10 years at a single sarcoma center.

## Patients and methods

### Patients

We performed a retrospective review of all patients who underwent sarcoma excision from 2004 to 2014 at our University Hospital. Inclusion criteria for this analysis were histologically confirmed diagnosis of soft tissue sarcoma of the extremities. A total of 182 patients with different entities of soft tissue sarcoma were treated with surgery. Of these, 49% were treated with neoadjuvant RCT and 7% with adjuvant RT, respectively. Indications for preoperative RCT were large tumors, close margins and to avoid R1-resection; for postoperative radiotherapy, higher stage diseases, dedifferentiated tumors and close margins. Neoadjuvant radiotherapy was only applied as RCT. Postoperative RT was applied without chemotherapy. Patients with neoadjuvant and adjuvant radiotherapy (1%, data not shown) as well as Patients treated with isolated hyperthermic limb perfusion were excluded, because of the very low number of patients treated that way rendering statistical analyses impossible. Data concerning patient characteristics, clinical variables, disease staging, and treatment outcomes were collected from the patient files and double-checked. There is a lack of data regarding precise tumor size in the majority of patients. However, size of excision is part of the data set recorded, so it was evaluated for this study as follows: we differentiated the median excision size into two groups: smaller than 10 cm or 10 cm and larger. For median size of excision, we analyzed the excision size in three diameters (length, width and height). From these data, we calculated the mean diameter and called it median size of excision.. Major complications are defined as complications which made an operative or in-hospital treatment necessary (Clavien-Dindo ≥3). Operations and in-hospital treatments were counted (1, 2, 3, > 3). Tumor staging was performed according to the TNM classification and the FNCLCC Grading (G1–3) [9]. QoL questionnaires were sent to all included patients and, if answers to any of the given questions were missing, questionnaires were completed by telephonic interview.

### QLQ measures

The HRQoL was assessed by using the core questionnaire of the European Organization for Research and Treatment of Cancer (EORTC QLQ-C30). The QLQ-C30 contains a global QoL scale, five function scales (physical, role, cognitive, emotional, and social), three symptom scales (fatigue, pain, and nausea/vomiting), and six single items (dyspnea, insomnia, appetite loss, constipation, diarrhea, and financial difficulties). All scores were linearly transformed such that they ranged from 0 to 100, in accordance with the EORTC Scoring Manual. A higher global QoL/health score equates to better overall QoL, and a higher score for functional scales corresponds to a better functioning, whereas higher score for a symptom scale indicates more symptoms [10]. HRQoL data about patients with adjuvant radiotherapy were excluded because of the small group size (3 patients).

### Statistics

Calculations were performed using the Statistical Package for the Social Sciences (version 19.0, SPSS Inc., Chicago, IL, USA).

**Fig. 1:**
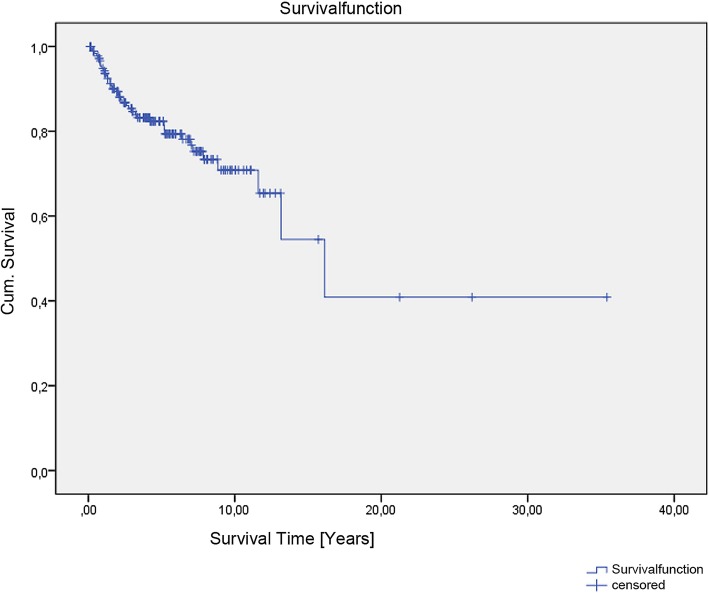
Kaplan-Meier Curve of Survival Function of the 182 patients with Extremity STS

Survival, including possible influencing factors, was calculated using the Kaplan–Meier method with log-rank tests (Mantel–Cox) (Fig. 1). Comparison of patient characteristics between the groups was performed by cross-tables and exact chi-square test, exact Mann–Whitney test, exact Fisher-test, and t-test for categorical, ordinal, and continuous variables, respectively. QoL data are presented as mean values and 95% confident interval. Comparisons between QoL scores for the groups were made by Shapiro-Wilk test and absent standard distribution by Wilcoxon test.

Additionally, LQ-Patients were stratified into four groups: Neoadj. RT or No RT and Disease-free, Neoadj. RT or No RT and Recurrence/Metastasis (further differentiation into a separate group recurrence and another group metastasis was because of the very low number of patients with recurrence or metastasis statistical impossible) and statistical significance were calculated using Kruskal-Wallis H-test and the subsequent pairwise comparison was performed using the Mann-Whitney U-test after Bonferroni procedure.

## Results

From 2004 to 2014, a total of 182 patients underwent sarcoma resection of the extremities in the Department of Surgery or the Department of Plastic and Hand Surgery of our University Hospital. Liposarcoma was the primary diagnosis, followed by undifferentiated pleomorphic sarcoma (UPS) including synovial sarcoma and fibroblastic/myofibroblastic sarcoma. The distribution of the different sarcoma subtypes treated at our institution is illustrated in Table 1.

**Table 1:** The proportions of the different sarcoma subtypes of all patients who underwent sarcoma excision from 2004 to 2014 in our University Hospital and of patients who answered life-quality quetionnaire. Subtypes were classified according to WHO classification

	All Patients *n* = 182	LQ-Patients *n* = 70
Liposarcoma	36 (20%)	16 (21%)
Well differentiated	21 (11,5%)	11 (16%)
De- differentiated	2 (1%)	2 (3%)
Myxoid	10 (5%)	2 (3%)
Pleomorph	3 (2%)	1 (1%)
Undifferentiated pleomorphic sarcoma	26 (14%)	7 (10%)
Synovial sarcoma	11 (6%)	5 (7%)
Fibroblastic/myofibroblastic sarcoma	43 (24%)	23 (30%)
Undifferentiated sarcoma	33 (18%)	9 (12%)
Leiomyosarcoma	13 (7%)	7 (9%)
Rhabdomyosarcoma	7 (4%)	2 (3%)
Extraskeletal chondro−/ osteosarcoma	5 (3%)	2 (3%)
Malignant peripheral nerve sheath tumor	4 (2%)	3 (4%)
Angiosarcoma	2 (1%)	0 (0%)
Other unclassified sarcoma	2 (1%)	1 (1%)

The overall survival rate after 5 years was 82% and after 10 years 71% (Fig. 1). The average age of the patients at the time of primary diagnosis of sarcoma was 58 years (range, 15–89), and the median follow-up time was 3.7 years. The majority of primary sarcoma was high-grade tumors (G2: 35%, G3: 43%). At definite operation on the primary sarcoma resection, in 89% complete resection was achieved with free surgical margins. In 8%, the tumor was macroscopically removed, but histopathological evaluation revealed an R1-status. In four cases (2%) only tumor mass debulking (R2) was performed. 49% of all included soft tissue sarcoma patients were additionally treated with neoadjuvant RCT and adjuvant RT was used in 7%. A summary of the data is given in Table 2.

**Table 2:** Patient and disease characteristics of 182 patients with soft tissue sarcoma which underwent sarcoma resection our University Hospital (All Patients) and of patients who answered life-quality questionnaire (LQ-Patients)

	All Patients				LQ-Patients		
	total	No RT	Neoadjv. RCT	Adjv. RT	total	No RT	Neoadjv. RCT
N =	182	75 (41%)	89 (49%)	13 (7%)	70	31 (44%)	39 (56%)
Median Age at first diagnosis [years]	58	59	59	54	57	56	60
Grading							
G1	36 (20%)	36 (48%)	4 (4%)	–	18 (25%)	18 (59%)	4 (9%)
G2	64 (35%)	23 (30%)	34 (38%)	2 (18%)	26 (37%)	8 (27%)	16 (42%)
G3	78 (43%)	17 (22%)	51 (57%)	9 (73%)	25 (35%)	4 (14%)	19 (49%)
Medium size of excision							
< 10 cm	104 (57%)	48 (64%)	45 (51%)	11 (82%)	43 (61%)	23 (73%)	19 (50%)
≥ 10 cm	78 (43%)	28 (37%)	44 (49%)	2 (18%)	27 (39%)	8 (27%)	19 (50%)
Localization							
Lower Extremity	137 (75%)	54 (72%)	70 (79%)	8 (62%)	51 (73%)	20 (65%)	31 (80%)
Upper Extremity	45 (25%)	21 (28%)	19 (21%)	5 (39%)	19 (27%)	11 (35%)	8 (20%)
5-year Survival rate	149 (82%)	68 (90%)	68 (76%)	10 (77%)	no calculation due to censored data		
10-year Survival rate	129 (71%)	62 (83%)	61 (68%)	–			
Local recurrence	28 (15%)	19 (10%)	7 (4%)	2 (1%)	6 (9%)	5 (16%)	1 (3%)
Metastasis	42 (23%)	12 (7%)	26 (14%)	4 (2%)	6 (9%)	2 (7%)	3 (8%)

Postoperative major complication rates (Clavien-Dindo ≥3) are presented in Table 3. After neoadjuvant RCT, a major complication rate of 28% vs. 7% without RT (*p* <  0.001) was observed. Major complications after adjuvant RT occurred in 8% with no significant difference to patients without RT (7%, not statistically significant, *p* = 0.265). 93% of patients with adjuvant RT had no major complications vs. 72% who had received neoadjuvant radiotherapy. However, in case of any major complications after adjuvant RT we noticed a minimum of two complications (vs. mostly one complication of patients with neoadjuvant radiotherapy). Major complications after chemotherapy (CT) only were not detected.

**Table 3:** Major-complications (Clavien-Dindo ≥3) of 182 patients with soft tissue sarcoma who underwent sarcoma resection

Major-complications	no RT (*n* = 75)	neoadjuvant RCT (*n* = 89)	adjuvant RT (*n* = 13)
No	69 (93%)	63 (72%)	12 (92%)
Yes	5 (7%)	25 (28%)	1 (8%)
Necrosis		8	
Wound healing disorders and Infections	4	9	
Thrombosis	1	1	1
Bleedings		3	
Other		4	
*p*-value (Fisher-test)		< 0.001^a^	0.169^b^
		0.265^c^	

^a^ neoadjuvant vs. no radiotherapy; ^b^ adjuvant vs. no radiotherapy; ^c^ neoadjuvant vs. adjuvant radiotherapy

**Fig. 2:**
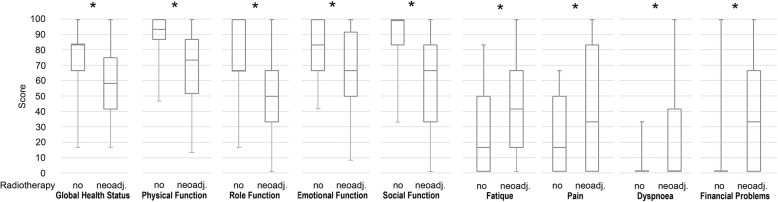
Quality of life scores of extremity soft tissue sarcoma patients who underwent preoperative RCT and surgery or no RT (and only surgery). x-pivot: 95% Confidence Interval of Quality of Life score. Global: global quality of life score; physical: physical functioning score; role: role functioning score; emotional: emotional functioning score; social: social functioning score; fatigue: fatigue score; dyspnoea: dyspnoea score; pain: pain score. Functioning scores: best score: 100. Pain Score: best score: 0. Figure showing only the significantly different scores/items

Seventy patients answered the QoL-questionnaires and were included in the analyses. 23% of the patients died, 27% gave no feedback and 12% of the patients refused to attend. Median time between treatment and questionnaires was 65 months (95% Confidence 59–72 months). All included patients reported that they fully understood the questionnaires. 56% of the patients were treated with neoadjuvant RCT and 44% of the patients were not irradiated. Comparison of QoL scores in the QLQ-C30 between the two groups with or without neoadjuvant RCT revealed significant differences in global QoL, in physical functioning, in role functioning, emotional functioning, social functioning, in fatigue, general pain and in financial problems (Table 4 and Fig. 2).

**Table 4:** Comparing quality of life in two different groups of patients with neoadjuvantor without radiotherapy

QLQ-C30	Radiotherapy		*p*-value (no radio-therapy vs. neoadjuvant)
	no	neo-adjuvant	
	(*n* = 31) 42%	(*n* = 39) 52%	
Global QoL score	73.1 (65–81)	58.6 (51–66)	0.006^*^
Physical function score	88.1 (83–94)	68.2 (60–76)	< 0.001^*^
Role function score	75.2 (67–84)	52.9 (43–63)	0.002^*^
Emotional function score	80.1 (73–87)	65.3 (57–74)	0.022^*^
Cognitive function score	88.8 (83–95)	80.1 (72–88)	0.247
Social function score	87.6 (80–95)	60,9 (51–71)	< 0.001^*^
Fatigue score	25.9 (15–37)	43.4 (34–53)	0.023^*^
Pain score	22 (13–31)	42.9 (31–55)	0.016^*^
Insomnia score	19.3 (10–29)	25,4 (15–36)	0.505
Appetite loss score	3.2 (−0.4–6.9)	3.9 (0.47–7.2)	0.68
Nausea and vomiting score	1.6 (−0–3.5)	1.7 (− 0.3–3.8)	0.824
Constipation score	6.4 (−1.5–14)	12.2 (4–21)	0.151
Diarrhoea score	6.4 (0.6–12)	10.8 (4.3–17)	0.286
Dyspnea score	7.5 (2.3–13)	22.8 (13–33)	0.034^*^
Financial problems score	11.8 (2–22)	31.5 (20–44)	0.009^*^

^*^ statistically significant (*p* < 0.05). Shown as CI = Confidence interval 95% and lower/upper confidence bounds (CI (lower-upper))

Stratification of Disease-control of the LQ-Patients into four groups (Neoadj. RT or No RT and Disease-free, Neoadj. RT or No RT Recurrence/Metastasis) showed significant differences in physical functioning (pairwise comparison: Neoadj. RT + Disease-free vs. No RT + Disease free, H = 17.979, *p* = 0.003*), role functioning (pairwise comparison: Neoadj. RT + Disease-free vs. No RT + Disease free, H = 13.440, *p* = 0,05*), social functioning (pairwise comparison: Neoadj. RT + Disease-free vs. No RT + Disease free, H = 17.904, *p* = 0.002*) and in dyspnea (pairwise comparison: Neoadj. RT + Disease-free vs. Neoadj. RT + Recurrence/Metastasis, H = − 25.588, *p* = 0.02*; Neoadj. RT + Recurrence/Metastasis vs. No RT + Recurrence/Metastasis, H = − 28.250, *p* = 0.05*; and, Neoadj. RT + Recurrence/Metastasis vs. No RT + Disease-free, H = − 32.250, *p* = 0.002*) and a strong trend in global QoL (H (3) = 7.572, *p* = 0.056) and in financial problems (H (3) = 7.567, *p* = 0.056) (Table 5).

**Table 5:** Stratification of Disease-Control (Disease-free vs. Recurrence/Metastasis). Analysis with Kruskal-Wallis H-Test. Values given as Mean Rank

QLQ-C30	No RT, Disease-free	No RT, Recurrence / Metastasis	Neoadj. RT, Disease-free	Neoadj. RT, Recurrence / Metastasis	H- and, *p*-value
Global QoL score	42.96	42.33	30.10	25.88	H (3) = 7.572 *p* = 0.056
Physical function score	44.92	44.33	26.94	27.50	H (3) = 13.787 *p* = 0.003^*^
Pairwise comparison			H- and *p*-value		
Neoadj. RT + Disease-free vs. No RT + Disease free			H = 17.979, *p* = 0.003^*^		
Neoadj. RT + Disease-free vs. Neoadj. RT + Recurrence/Metastasis			H = 0.559, *p* = 1.00		
Neoadj. RT + Disease-free vs. No RT + Recurrence/Metastasis			H = 17.392, *p* = 0.28		
Neoadj. RT + Recurrence/Metastasis vs. No RT + Recurrence/Metastasis			H = 16.833, *p* = 1.00		
Neoadj. RT + Recurrence/Metastasis vs. No RT + Disease-free			H = 17.420, *p* = 0.616		
No RT + Recurrence/Metastasis vs. No RT + Disease-free			H 0.587, *p* = 1.00		
Role function score	43.44	45.17	30.00	19.50	H (3) = 10.696 *p* = 0.01^*^
Pairwise comparison			H- and *p*-value		
Neoadj. RT + Disease-free vs. RT + Disease free			H = 13.440, *p* = 0,05^*^		
Neoadj. RT + Disease-free vs. Neoadj. RT + Recurrence/Metastasis			H = 10.500, *p* = 1.00		
Neoadj. RT + Disease-free vs. No RT + Recurrence/Metastasis			H = 15.167, *p* = 0.50		
Neoadj. RT + Recurrence/Metastasis vs. No RT + Recurrence/Metastasis			H 25.667, *p* = 0.27		
Neoadj. RT + Recurrence/Metastasis vs. No RT + Disease-free			H = 23.940, *p* = 0.15		
No RT + Recurrence/Metastasis vs. No RT + Disease-free			H = − 1.727, *p* = 1.00		
Emotional function score	40.88	44.92	31.73	20.75	H (3) = 6.468 *p* = 0.09
Cognitive function score	37.46	39.83	33.24	28.38	H (3) = 1.668 *p* = 0,64
Social function score	45.58	42.83	27.68	19.38	H (3) = 16.108 *p* = 0.001^*^
Pairwise comparison			H- and *p*-value		
Neoadj. RT + Disease-free vs. RT + Disease free			H = 17.904, *p* = 0.002^*^		
Neoadj. RT + Disease-free vs. Neoadj. RT + Recurrence/Metastasis			H = 8.301, *p* = 1.00		
Neoadj. RT + Disease-free vs. No RT + Recurrence/Metastasis			H = 15.157, *p* = 0.45		
Neoadj. RT + Recurrence/Metastasis vs. No RT + Recurrence/Metastasis			H = 23.458, *p* = 0.35		
Neoadj. RT + Recurrence/Metastasis vs. No RT + Disease-free			H = 26.205, *p* = 0.06		
No RT + Recurrence/Metastasis vs. No RT + Disease-free			H 2.747, *p* = 1.00		
Fatigue score	27.50	24.42	36.57	44.38	H (3) = 5.969 *p* = 0.11
Pain score	28.52	29.92	39.09	48.38	H (3) = 6.598 *p* = 0.08
Insomnia score	33.42	33.25	33.79	57.75	H (3) = 6.665 *p* = 0.08
Appetite loss score	35.20	31.00	36.03	31.00	H (3) = 1.589 *p* = 0.66
Nausea and vomiting score	34.72	37.67	34.09	40.50	H (3) = 2.020 *p* = 0.56
Constipation score	33.30	29.00	35.93	46.75	H (3) = 4.962 *p* = 0.17
Diarrhoea score	32.20	33.67	35.11	45.12	H (3) = 2.946 *P* = 0.40
Dyspnea score	29.50	33.50	36.16	61.75	H (3) = 13.168 *p* = 0.004^*^
Pairwise comparison			H- and *p*-value		
Neoadj. RT + Disease-free vs. RT + Disease free			H = −6.662, *p* = 0.78		
Neoadj. RT + Disease-free vs. Neoadj. RT + Recurrence/Metastasis			H = −25.588, *p* = 0.02^*^		
Neoadj. RT + Disease-free vs. No RT + Recurrence/Metastasis			H = − 2.662, *p* = 1.00		
Neoadj. RT + Recurrence/Metastasis vs. No RT + Recurrence/Metastasis			H = −28.250, *p* = 0.05^*^		
Neoadj. RT + Recurrence/Metastasis vs. No RT + Disease-free			H = − 32.250, *p* = 0.002^*^		
No RT + Recurrence/Metastasis vs. No RT + Disease-free			H = − 4.000, *p* = 1.00		
Financial problems score	28.42	30.83	39.21	46.62	H (3) = 7.567 *p* = 0.056

^*^ statistically significant (*p* < 0.05). Pairwise Comparison using Mann Whitney U-Test after Bonferroni procedure

## Discussion

In this study we present our results of soft tissue sarcoma patients QoL in a retrospective single-center study and tried to differentiated into different groups in case of radio (chemo) therapy, compared to non-irradiated patients.

Many other retrospective studies, register studies and systematic reviews have examined the role of neo- and adjuvant radio (chemo) therapy in regard to different parameters apart from QoL, such as local control, recurrence rate and overall survival, for which the quality of surgical resection seems to be crucially [11, 12]. Since the first description of Rosenberg [1], the combination of surgery and radiotherapy in soft tissue sarcoma treatment is well established [13]. Al-Absi et al. concluded that delayed surgical resection because of preoperative radiation does not seem to increase the risk of lethal metastatic spread [5]. To date, no differences were found in overall survival, progression free, or local disease control in case of radiotherapy in soft tissue sarcoma treatment. However, recently a meta-analysis suggested that radiotherapy is associated with lower long-term mortality [14]. In our patient population, 49% of all included patients were additionally treated with neoadjuvant RCT. Other studies reported much lower radiation rates ranging from 9% in contrast to much higher rates up to 100% for planned sarcoma excision [15, 16].

In our study, we first investigated postoperative major complications of pre- and postoperative radio (chemo) therapy in the treatment of soft tissue sarcoma. In each case, we analyzed the kind of major complication and classified it to postoperative major complication rate or not. In the case of postoperative RT and postoperative major complications, the time period between surgery and RT were very close or classification clear. Major and overall complication rates were not significantly different in one study (major: 28.2 vs. 25.2%, *p* = 0.69; overall 35.2 vs. 33.2%, *p* = 0.83) [15]. However, we observed statistically significant differences in major complications rates after neoadjuvant RCT (28 vs. 7%, *p* < 0.001), but not after CT only (0 vs. 7%, *p* = 0.13). The reason for differences in complication rates of irradiated patients remains unclear. Nussbaum et al. reported on a very large study population (785 Patients) with a small radiation rate of 9% (our data 49%) and focused on short-term (30 days) morbidity and mortality as well as on retroperitoneal sarcoma [15]. Meric et al. found a 50% increase in wound complications among patients treated with preoperative RT [17]. In case of postoperative RT, we noticed major complication rates of 8% (vs. 7%, *p* = 0.265). In accordance with our results, Miller et al. reported higher wound complication rates after neoadjuvant than after postoperative RT (35 vs. 17%, *p* = 0.01) [18]. In a prospective trial by O’Sullivan et al., 190 patients with extremity soft tissue sarcoma were randomly allocated to either preoperative or postoperative RT. Acute wound complications were significantly higher in the neoadjuvant group (35 vs. 17% in the postoperative group) [19].

Major complication rates which need additional operations or in-hospital treatment are serious adverse events. Even though the levels of these complication rates are in accordance with the literature, all efforts should be made to further decrease those levels. However, the risk of such major complications may not only be related RT, but possibly even more to the patient [20] and individual tumor characteristics [21] and tumor specific molecular mechanisms [22, 23]. In addition, different surgical resection modes (wide excision or compartment resection in contrast to simple resection [24]), differences in preoperative treatments, and various localizations (maybe affecting of neurovascular structures bones or joints) of soft tissue sarcomas may play a role in major complication rates. Furthermore, specific technical RT parameters such as total dose, fraction size, treatment volume and RT techniques, which could not be analyzed within this study due to incomplete data, may play a pivotal role for the risk of developing major complications.

Our study also had some additional limitations according to other investigations in the literature [25]. First, we included 182 patients with different tumor localizations, different clinical and/or pathological statuses and various soft tissue sarcoma subtypes, because individual subtype analyses would have resulted in very small group sizes, rendering statistical analysis impossible [26]. In general, the larger and more malignant a STS is, the more likely the patient is to receive chemotherapy and/or radiotherapy. Conversely, small superficial tumors are unlikely to receive CT or RT. In contrast to a prospective analysis, retrospective data analysis allows no stratification for example regarding tumor size. Possibly prospective studies with stratification regarding tumor size (and/or grading) might show that the very tumors which did not need chemotherapy or RT are at the same time more eligible to complete surgical resection and thus might result in quicker recovery with less complications and as a consequence yielding better QoL. Furthermore there are well-recognized limitations of retrospective studies per se, e. g. dependence on the limited medical data records in particular concerning patients who are deceased or lost to follow-up, no measurement of late toxicity effects and selection bias [27]. Since patients were contacted after completion of their therapy (median interval: 4.98 years), we were only able to report final outcome QoL not during the mean time / whilst being on therapy, which might also influence the final and ex post QoL estimation by the patients. Furthermore, because of the retrospective analyses we have a lack of baseline evaluation of QoL before treatment with or without radio (chemo)therapy. It is possible, that patient candidate to radiation therapy could have had a worse QoL baseline due to more advanced disease. Finally, the sample size of this series still is not very large, with is attributed to the rarity of this disease. Nevertheless, given the lack of literature on QoL outcomes after radio (chemo) therapy and the rarity of these tumors, we hope this study might add new information regarding the possible impact of neo−/adjuvant radio (chemo) therapy on QoL in soft tissue sarcoma patients, even it is not possible to discriminate whether radiation therapy could really had an impact or not in retrospective studies.

To our best knowledge there are only a few investigations of QoL in soft tissue sarcomas [28]: the purpose of the study by Parsons et al., e. g., was to investigate rehabilitation aims of patients with soft tissue sarcoma and chronic disability using the World Health Organization’s (WHO) international classification of functioning, disability, and health (ICF). There was the strongest support for complex decongestive physiotherapy and aerobic exercise interventions [29]. In 2006 Schreiber et al. evaluated function- and health-related quality of life in 100 extremity soft tissue sarcoma patients, using life orientation test (LOT), musculoskeletal tumor society rating scale (MSTS), reintegration to normal living index (RNL), and Toronto extremity salvage score (TESS). Restriction in participation of life roles and situations has the greatest effect on these patients [30]. Patients’ QoL (investigated with RAND-36) after hyperthermic isolated limb perfusion for locally advanced extremity soft tissue sarcoma was significantly worse in physical functioning comparing to the healthy Dutch population [8]. Investigations of QoL after compartimental resection for subfascial extremity soft tissue sarcoma (using EORTC Score C30) showed decreased QoL scores in all dimensions, compared to a normal population [31]. Reichardt et al. and Coens et al. investigated the HRQoL of patients with soft tissue sarcoma and chemotherapy [32, 33]. Sachsenmaier et al. created a new questionnaire. Based on this questionnaire, they were able to identify risk factors for poor emotional outcome after therapy, related to patients’ physical, psychological and social situation [34]. Xu et al. reported in 2017 a better functional outcome and QoL using Chinese MSTS scoring system for patients receiving limb-salvage surgeries than those undergoing amputation surgeries [35].

Overall, literature about QoL of patients with soft tissue sarcoma and radiotherapy is very rare. To the best of our knowledge there have been no HRQoL investigations of patients with soft tissue sarcoma and radiotherapy (except hyperthermic limb perfusion) reported in the literature until today. Some studies address the functional outcome after surgery of extremity soft tissue sarcomas in scores, but the scales vary between the reports. A review of 145 patients which were treated with limited surgery and postoperative radiotherapy showed that 20% of patients developed contracture, 19% significant edema, 7% required the use of a crutch, and 6% experienced a bone fracture [36].

The EORTC QLQ-C30 used in this study is well established and validated in cancer [10, 37]. It is multidimensional, incorporating all aspects of daily life, as well as being subjective [38]. Comparing the group of preoperative RCT vs. non-irradiated patients shows that global QoL, physical, role, and social functioning, emotional functioning, fatigue, pain and financial problem scores were significantly worse in the neoadjuvant radiotherapy group, compared to the group without RT (Table 4 and Fig. 2). It is known that RT in cancer therapy affected cancer patients’ QoL negatively [39]. In contrast to Yucel et al. [39], we did not measure a restoration of pre-treatment HRQoL after completion off the RT. This is in accordance with the study of Bansal at al., evaluating 45 patients with head and neck cancer [40, 41]. After completion of sarcoma treatment, we still observed significantly worse scores for global QoL, physical, role, and social functioning, emotional functioning, fatigue, pain and financial problems.

RT itself is administered over weeks. This is also a probable reason for financial difficulties, because patients were possibly far away from home and off work for a long time [38]. It is also possible that the combination of RT and intensive CT causes the reduced HRQoL scores. However, some studies have shown reduced QoL when radiotherapy and chemotherapy were combined compared to radiotherapy alone [42]. Further analysis of CT effects alone (without RT) would be helpful.

Disease status of the patients who answered the questionnaires could have been an influence of QoL. Stratification into four groups (No RT Disease-free, Neoadj. RT Disease-free, No RT Recurrence/Metastasis, Neoadj. RT Recurrence/Metastasis) found significant reduced scores of physical functioning, role functioning and social functioning in the group of disease-free patients with neoadjuvant RT compared to the disease-free patients without RT and a strong trend for global QoL and financial problems without statistical significance. A direct correlation of QoL to the initial treatment is despite these results difficult, because the number of patients living with recurrence, metastasis or disease-control was differently and a further differentiation between patients with recurrence and patients with metastasis was in our collective not possible.

Nevertheless, further analysis and especially prospective studies of QoL with stratifications of disease control in STS patients with and without radiotherapy are necessary to evaluate a directly correlation of treatment modalities in primary disease and QoL.

## Conclusion

We present our results of soft tissue sarcoma patients’ HRQoL in the case of RT in a retrospective single-center study. We observed statistically significant differences in major complications rates after neoadjuvant RCT (28 vs. 7%, *p* < 0.001). In the case of postoperative RT, we observed similar major complication rates as compared to no-RT (8% vs. 7%, respectively; *p* = 0.265). We observed that global QoL, physical, role, and social functioning, emotional functioning, fatigue, pain and financial problem scores were significantly worse in the neoadjuvant RCT group, compared to the group without RT. Given the lack of literature on HRQoL outcomes after RT in soft tissue sarcoma and the rarity of these tumors, we think this study may help to reduce this gap of knowledge, even there are limitations especially because of the retrospective analysis and a direct conclusion of the primary treatment and the QoL are not possible.

## Data Availability

The datasets used and analyzed during the current study are available from the corresponding author on reasonable request.

## Abbreviations

CT: Chemotherapy
EORTC: European Organization for Research and Treatment of Cancer
HRQoL: Health related quality of life
MSTS: Musculoskeletal tumor society rating scale
QoL: Quality of life
RCT: Radiochemotherapy
RNL: Reintegration to normal index
RT: Radiotherapy
STS: Soft tissue sarcoma
TESS: Toronto extremity salvage score
UPS: Undifferentiated pleomorphic sarcoma
WHO: World Health Organization
